# Optimizing extinguishing angle of 6MF-30 pneumatic extinguisher for wildland fire-fighting based on CFD and experiments

**DOI:** 10.1038/s41598-023-37275-x

**Published:** 2023-06-22

**Authors:** Fanbao Chen, Bin Miao, Qingbiao Wang

**Affiliations:** 1 College of Resources, Shandong University of Science and Technology, Tai’an, 271019 China; 2 National Engineering Laboratory for Coalmine Backfilling Mining, Shandong University of Science and Technology, Tai’an, 271019 China; 3 China University of Mining and Technology, Xuzhou, China

**Keywords:** Engineering, Mechanical engineering

## Abstract

The 6MF-30 pneumatic extinguisher is a widely utilized and efficient tool for combating wildland fires. However, using incorrect extinguishing angles can diminish its effectiveness. This study aimed to determine the optimal extinguishing angle for the 6MF-30 pneumatic extinguisher by conducting computational fluid dynamics simulations and experimental verification. The findings revealed that ground roughness did not significantly affect the optimal extinguishing angle or the attenuation of jet velocity near the fan outlet region. The study determined that an optimal extinguishing angle of 37° applies to lossless ground, natural grassland, grassland with artificial disturbance, and enclosed grassland. Furthermore, among the selected angles, the highest rate of jet velocity reduction was observed at 45°, whereas the slowest reduction occurred at 20° and 25°. These findings offer valuable insights and recommendations for enhancing the efficacy of wildland fire-fighting when employing the 6MF-30 pneumatic extinguisher.

## Introduction

Due to human activities and climate change, the forest area is experiencing a decline, while the frequency of wildfires is progressively increasing on an annual basis^1–3^. A report published by the United Nations Environment Programme states that even the most robust plans for curbing greenhouse gas emissions will be insufficient to avert a substantial rise in the occurrence of extreme fires. It warns that wildfire events similar to Australia’s 2019–2020 black summer or the 2020 Arctic fire could witness an escalation of 31–57% by the end of the century^4^. The escalating anthropogenic activities and wildfire hazards have resulted in the proliferation of seedlings and monoculture within forest plant communities. This phenomenon has diminished the stability of forest ecosystems, exerting a detrimental impact on the global environment^5,6^.

**Figure 1:**
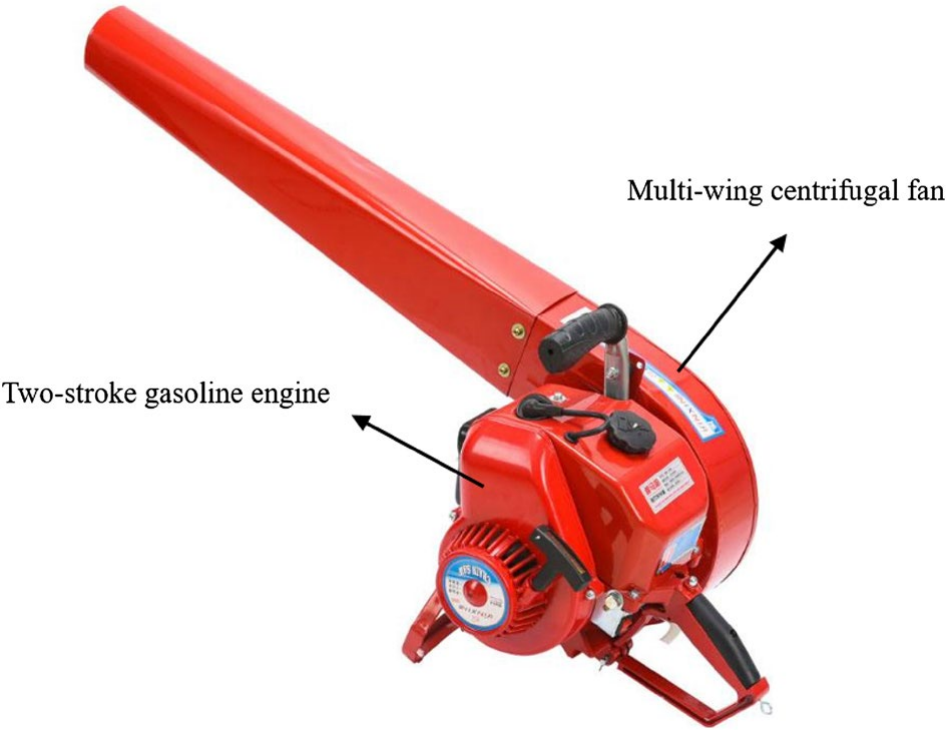
The pneumatic extinguisher.

Presently, wildfire suppression in the majority of China’s complex terrain areas continues to rely predominantly on manual firefighting techniques employing portable firefighting tools. For instance, in the forested regions of southwestern China, where the risk of forest fires escalates annually, the terrain predominantly comprises intricate topography with low road density, rendering it challenging for large fire-fighting vehicles to reach the fire scene and extinguish the flames. Aerial firefighting methods, such as the utilization of aircraft and artificial rainfall, are both expensive and subject to limitations^7–10^. Portable fire extinguishing devices are the optimal choice for forest fire departments operating in this region. The pneumatic extinguisher offers several advantages, including simplicity of operation, cost-effectiveness, and portability. During the fire extinguishing process, the high-speed air stream effectively disperses volatile matter away from the gas phase combustion reaction area. Simultaneously, the strong air stream significantly dilutes the concentration of combustible gas, ultimately impeding the gas phase combustion reaction when the velocity reaches a sufficiently high level. Consequently, the surface combustion area fails to receive a stable heat supply, leading to a sharp decline in the pyrolysis reaction rate and a continuous reduction in the concentration of combustible gas until the open flame dissipates. In comparison to other portable fire-fighting tools, the pneumatic extinguisher demonstrates superior fire extinguishing efficiency, making it one of the primary fire-fighting equipment utilized in China's forests and grasslands. Furthermore, the 6MF-30 pneumatic extinguisher (Fig. 1) stands as the pioneering fire extinguishing equipment employed specifically for forest and grassland firefighting in China. This device effectively suppresses fires by generating high-speed airflow through a two-stroke gasoline engine. It finds extensive utilization and maintenance within numerous forest and grassland fire extinguishing teams across China, serving as an indispensable asset for fulfilling the mission of forest and grassland fire suppression^11–14^.

Pneumatic extinguishers are categorized as centrifugal fans. While there are limited simulations conducted by international scholars on the flow field generated by this type of extinguisher, similar centrifugal fans can serve as a reference point. Hariharan and Govardhan conducted computations on an industrial centrifugal blower using the CFD commercial software CFX. They employed the *k–ε* two-equation model as the turbulence model^15^. Madhwesh et al. also employed *k–ε* two-equation model to assess the performance of a swept-back centrifugal fan. The calculation of the relative flow within the system was carried out using the sliding mesh method, thereby offering a crucial point of reference for this study^16^. Karanth and Sharma conducted CFD calculations to analyze the fluid flow characteristics within the region extending from the diffuser inlet to the impeller outlet. This analysis ultimately led to improvements in the dynamic head and static head of the fan^17^. Younsi et al.^18^ used the SST *k–ω* turbulence model to analyze the aerodynamic characteristics within a forward-swept centrifugal fan. However, in the study conducted by Petit and Nilsson, it was found that the standard k–ε turbulence model exhibited greater accuracy compared to the predictions made by the realizable *k–ε*, RNG *k–ε* and SST *k–ω* models^19^. However, there has been limited research conducted on the jet field of centrifugal fans. Chougule et al., conducted CFD simulations involving multiple air jets impacting flat plates, following a comprehensive evaluation of the findings from Thakare and Joshi, as well as Shi et al.^20–22^. The findings revealed that the SST *k–ω* model exhibited superior predictive capabilities for fluid flow and heat transfer characteristics. The study employed a structured mesh for jet calculations and applied mesh refinement on the near-wall surface, resulting in improved predictions of near-wall flow phenomena.

This paper presents a computational fluid dynamics simulation-based analysis of the internal and external flow fields of a specific model of pneumatic extinguisher. The investigation focuses on studying the rate decay characteristics of the jet field and the flow contour surface by varying the jet angle of extinguishers and the ground roughness. The findings of this study elucidate the effective fire extinguishing range of the analyzed fire extinguishing fan model and identify the optimal jet angle. These findings hold significant practical implications for firefighting operations and provide practical recommendations for grassroots forest fire prevention personnel.

## Methods

### Pre-processing and solver setup

**Figure 2:**
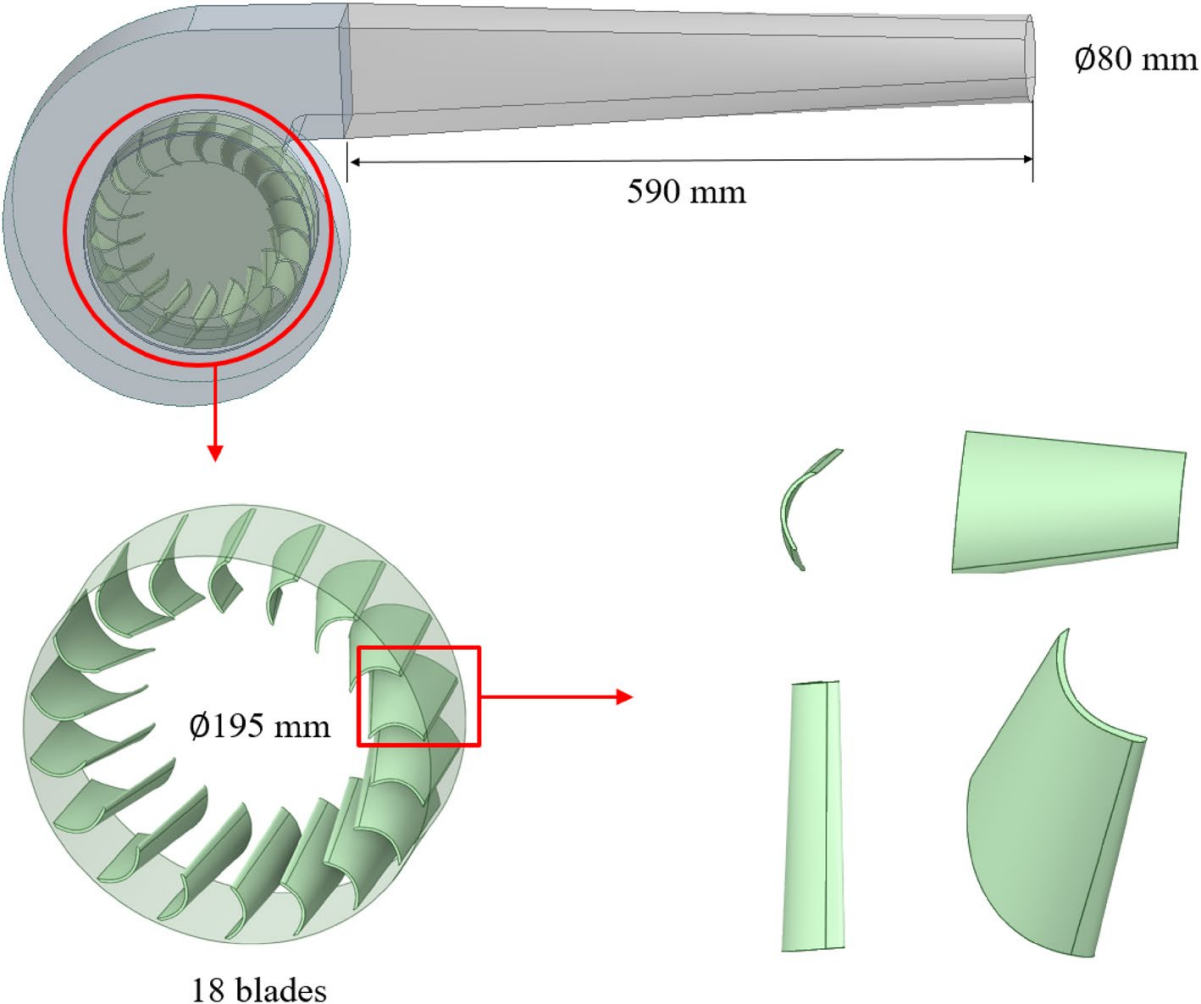
3D model of the pneumatic extinguisher.

**Figure 3:**
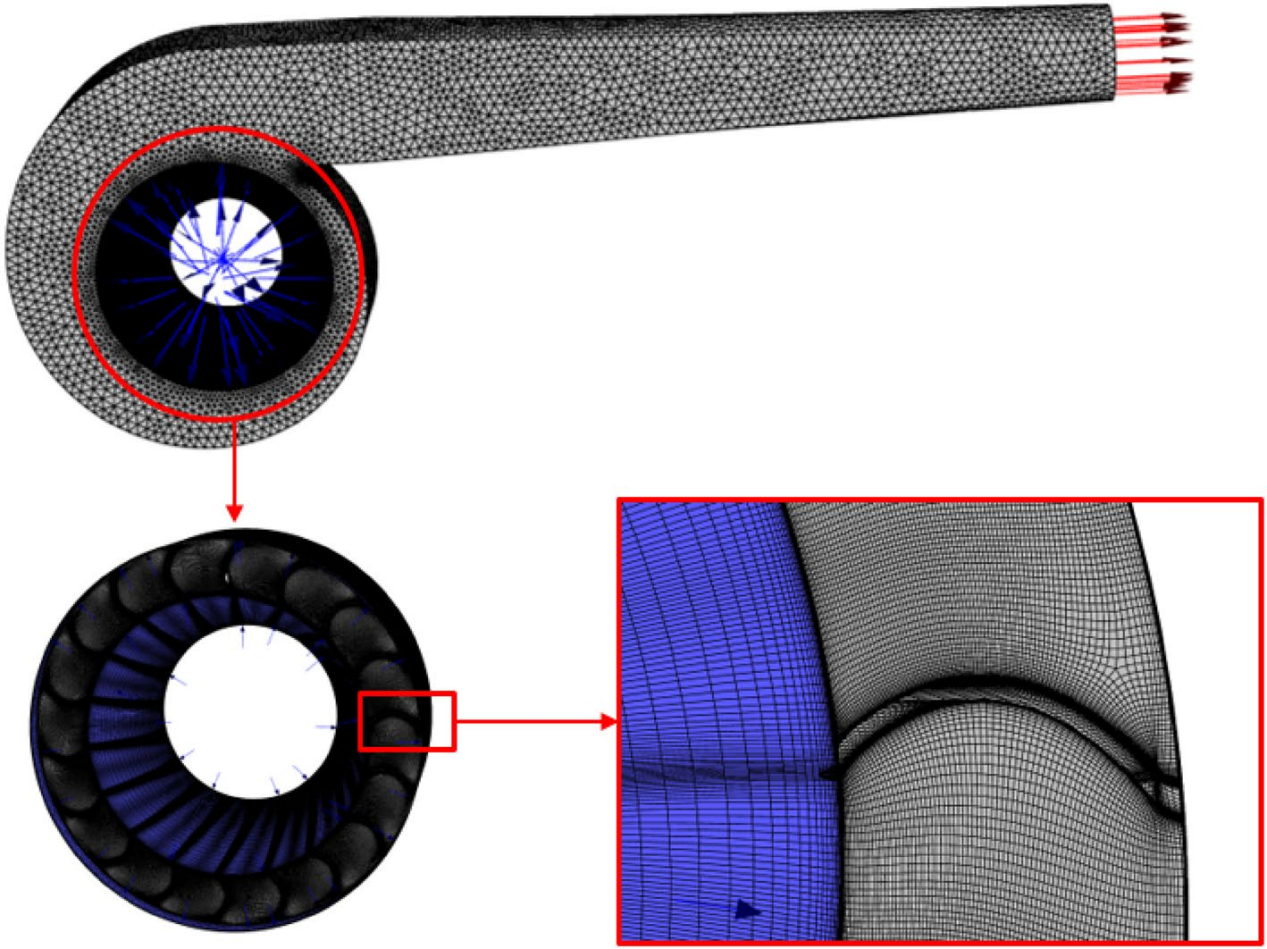
The internal mesh of the pneumatic extinguisher.

The computational solver utilized in this study is ANSYS Fluent (2020R2). Accurate modeling of the internal flow field area of the fan is necessary to calculate the fan jet field with a reference value. Figure 2 depicts the constructed 3D model of the internal flow field area, representing the internal domain of the extinguisher. For enhanced accuracy, the impeller rotation region is constructed with a structured grid and encrypted, while the remaining region is constructed with an unstructured grid, as depicted in Fig. 3.

The boundary conditions for the simulation are defined as follows: the inlet is specified as a pressure inlet, the outlet as a pressure outlet, and all wall boundaries as no-slip walls. In real flow scenarios, solid surfaces introduce resistance to the fluid due to wall friction. When the fluid interacts with a solid wall, collisions between fluid and wall molecules lead to velocity variations. Therefore, employing the no-slip wall condition, which assumes that the fluid velocity matches the wall velocity, effectively simulates the frictional interaction between the fluid and physical walls. The boundary layer corresponds to the region near a wall in a fluid flow where there is a substantial velocity gradient. Selecting the no-slip walls boundary condition allows for modeling the velocity distribution within the boundary layer and predicting key parameters like the boundary layer thickness and shear stress. This is vital for investigating flow characteristics in proximity to walls and predicting heat and mass transfer near wall surfaces. Thus, the choice of the no-slip walls boundary condition is both rational and essential for simulating fluid flows. It accurately represents wall friction, enables boundary layer modeling, accounts for flow resistance, and captures viscous effects near the wall^23,24^.

The mesh is partitioned into two regions: the dynamic region encompassing the impeller rotation region and the static region outside it. This division enables the simulation of the impeller rotation in the solver using the multiple reference frames (MRF) model and the sliding mesh method.

The MRF model is a simplified approach for multi-region calculations. It allows for the assumption of different rotational or translational velocities in various regions, treating the transient problem as a steady-state problem that requires solution. When a region remains stationary, the equations are converted into the stationary system form. A local reference system is employed at the boundary of each cell in the computational domain to precisely calculate the flow variable flux between neighboring cells. The reference system transfers the variables from one cell and applies them to the adjacent cell. Consequently, the flow variable values are accurately transferred and localized to each cell, facilitating precise calculations within the respective cell.

The sliding mesh method employs multiple computational regions, each capable of independent meshing, offering significant convenience for complex models. Each region intersects with at least one adjacent region. The intersection of the adjacent computational regions forms a mesh division, with the motion domain moving along the intersection. The grid at the intersection does not require alignment, and the flux is achieved through interpolation of information between nodes. A virtual grid layer is generated on each side of the sliding surface, overlapping with the computational domain grids on both sides. In addition, the nodes on the virtual grid layer interpolate to facilitate flux transfer between the computational domains on both sides of the intersection during computation.

**Figure 4:**
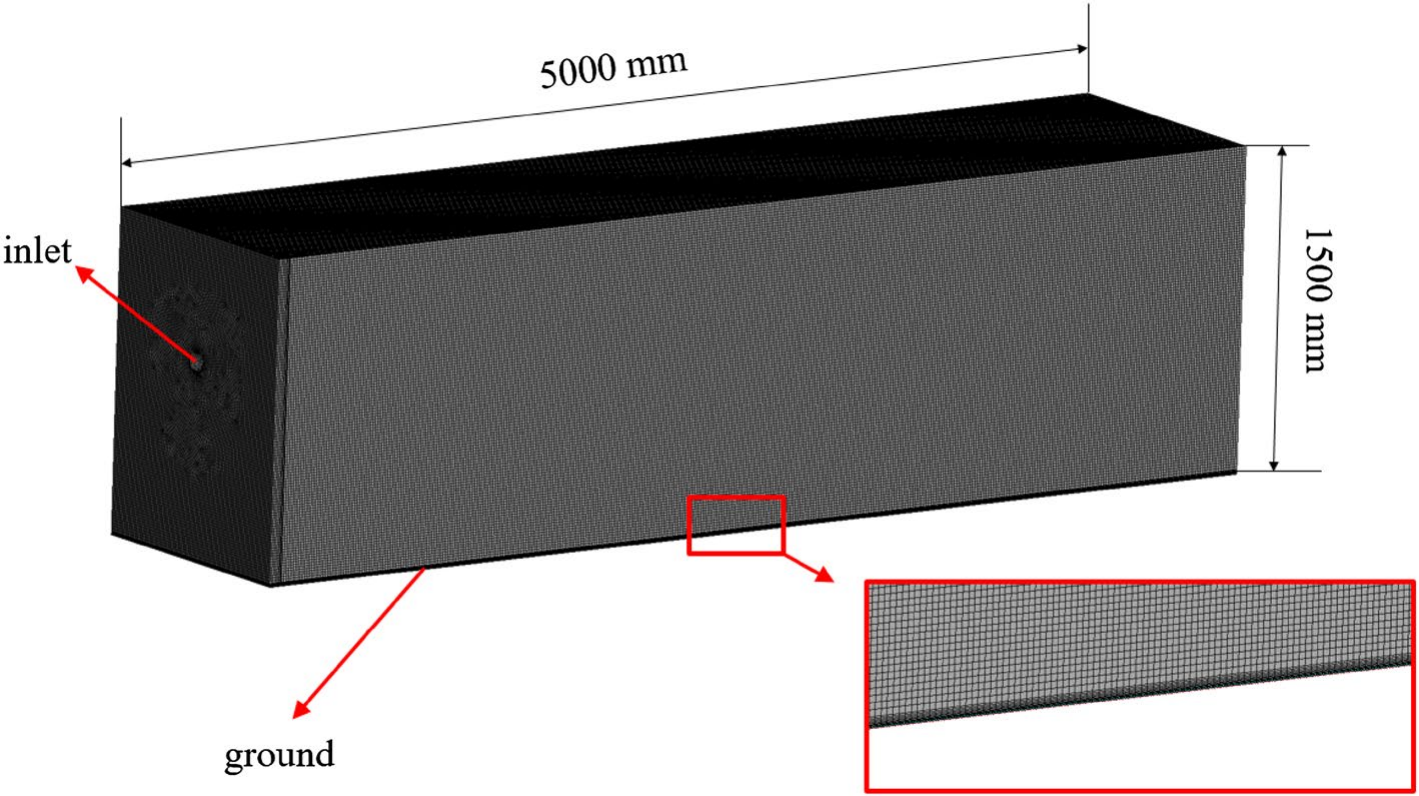
The external mesh of the pneumatic extinguisher.

To enhance computational accuracy, a comparative analysis will be carried out between the sliding mesh method and the MRF method, given their disparate solving strategies that can potentially produce varying calculation results. In order to improve calculation efficiency, the computational results obtained at the fan's outlet in the internal flow field are extracted and utilized as the inlet boundary conditions for the external flow field. The external flow field region of the fan lacks complex structures, enabling the utilization of a hexahedron-based mesh to enhance computational efficiency. The mesh file for this mesh type is illustrated in Fig. 4. Additionally, the inlet is designated as a velocity inlet, while the outlet is specified as a pressure outlet.

Commonly used turbulence models comprise the standard k–ε model, designed for high Reynolds number flows, the Realizable k–ε model, an improvement upon the former, and the RNG *k–ε* model, suitable for low Reynolds number flows^25–27^. Furthermore, the standard *k–ε* turbulence model finds wide usage in the field of flow analysis^28^. The SST k–ω turbulence model, which integrates the standard *k–ε* and standard *k-ω* models through a mixing function, yields more accurate calculation results in the jet region^29^. For specific equations, reference can be made to the theory guide of ANSYS Fluent^24^. Based on the preceding analysis, the standard k–ε turbulence model is chosen for the internal flow field of the fan to account for the high Reynolds number flows, while the SST k–ω turbulence model is employed for the jet region.

### The simulation scheme

**Figure 5:**
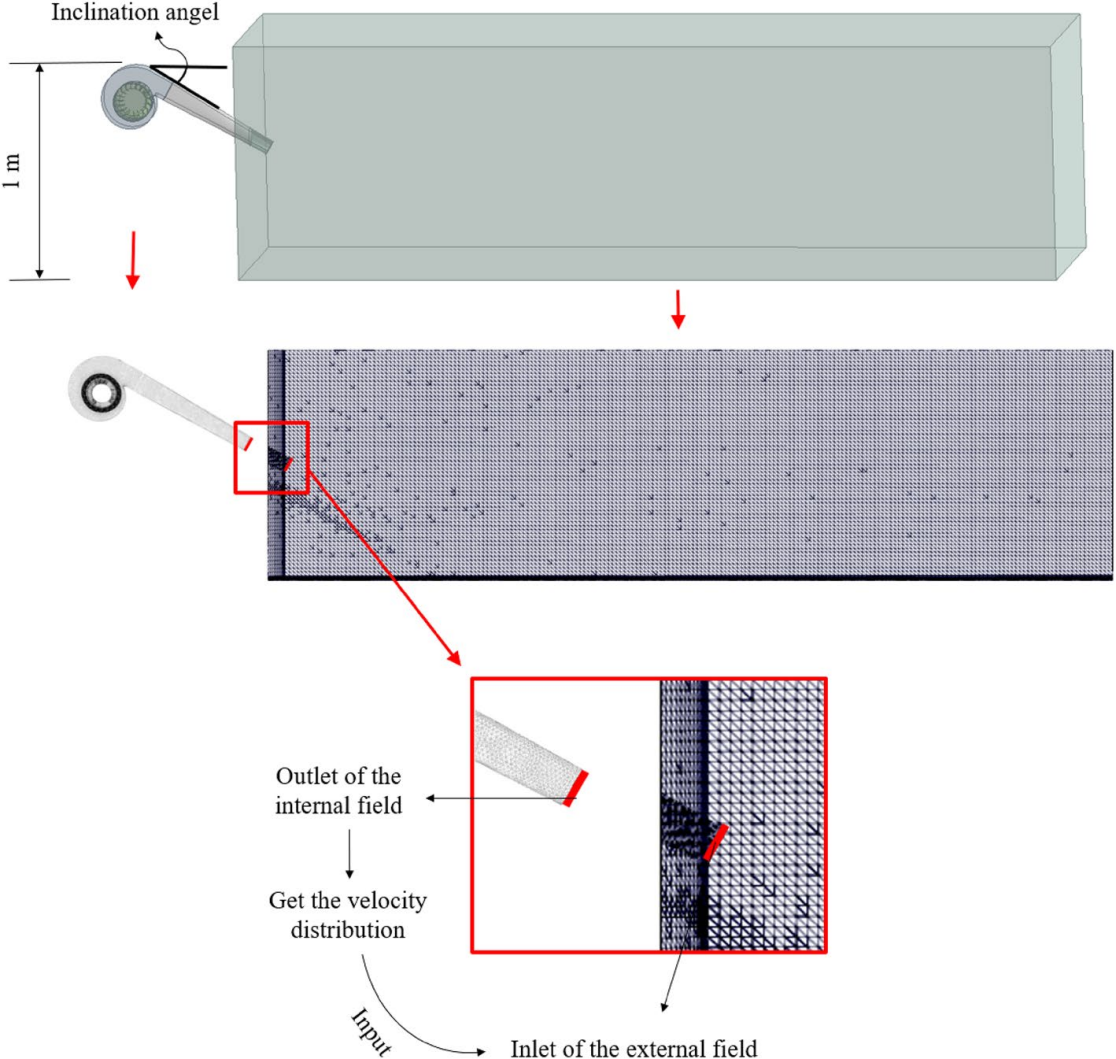
The simulation scheme.

The specific simulation scheme is shown in Fig. 5. During the modelling stage, the height of the 6MF-30 fan from the ground and the jet angle must be established, with the height determined based on practical experience at 1 m. The inclination angle represents the angle between the horizontal line and the center line of the tilted duct. The maximum jet angle of 45° is determined based on the distance from the fire source, which exceeds 1 m. Additionally, the minimum inclination angle was set to 20° based on experimental findings. When the inclination angle was below 20°, the jet distance became excessively long, resulting in low air speed near the ground, which deviated from the actual fire extinguishing operation. Jet angles ranging from 20° to 45°, spaced at equal intervals, were established to serve as the foundation for subsequent analysis.

Subsequently, the size of the external flow field area is modified based on the specific jet simulation results to ensure the acquisition of comprehensive velocity field information within the jet region. During the meshing stage, separate meshes were created for the two regions as depicted above, ensuring the alignment of the fan exit coordinates with the inlet of the external flow field. This process ensures the transfer of the velocity field at the fan outlet to the inflow boundary of the outflow field, enabling the utilization of different turbulence models for the two regions. During the calculation phase, the internal flow field of the fan is initially computed, and subsequently, the velocity distribution at the outlet is extracted. This data is then utilized as the input at the inlet of the outflow region prior to conducting calculations for the outflow field.

Additionally, the ground roughness of the domain will be assigned varying values based on the literature^30^. The roughness can be calculate by Eq. (1),

1 $$\ln Z_{0} = \frac{{\ln Z_{2} - \frac{{U_{2} }}{{U_{1} }}\ln Z_{1} }}{{1 - \frac{{U_{2} }}{{U_{1} }}}}$$

in which *Z*_0_ represents the ground roughness, *Z*_1_ and *Z*_2_ correspond to different altitudes, *U*_1_ and *U*_2_ indicate the airflow speed at *Z*_1_ and *Z*_2_, respectively.

## Mesh independence test and experimental verification

### Grid independence test

To mitigate the impact of mesh size on the calculation outcomes, it is necessary to conduct a grid independence check prior to analyzing the results. In this study, it is necessary to perform a mesh independence check for both the internal and external flow field meshes of the impeller.

The mesh of the flow field inside the extinguisher is divided into structured and unstructured grids. The structured grid area, which is more challenging to generate and has undergone meticulous encryption. The focus of the mesh independence check lies solely on the unstructured grid area, known as the stationary regionso. To conduct the check, the mesh size is progressively increased by a factor of two. Table 1 presents the mesh data corresponding to the maximum cell size. By analyzing the data in Table 1, the impact of the maximum cell size on the accuracy and reliability of the computational results can be evaluated. This examination plays a crucial role in ensuring the robustness of the numerical simulations and enhancing the overall quality of this research.

**Table 1:** The internal mesh data (unstructured).

Maximum cell size (m)	Number of nodes	Number of elements
0.002	863,745	2,948,333
0.004	242,194	840,669
0.008	110,442	432,240
0.016	85,789	361,904

**Figure 6:**
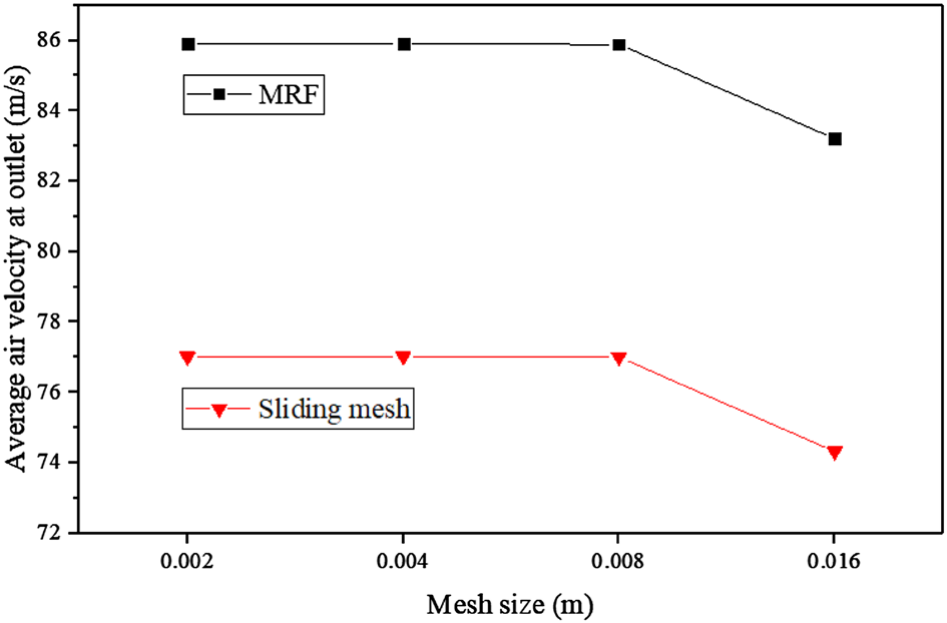
Comparison of the results calculated by MRF and sliding mesh in different mesh sizes (Internal flow field mesh).

During the calculation stage, both the MRF model and sliding mesh method were employed. The average outlet air speed served as the test parameter, while the fan speed remained fixed at 6300 r/min (rated speed). The corresponding calculation results are shown in Fig. 6. It can be observed from the figure that a significant decrease in the calculation results occurred as the mesh size increased to 0.016 m. Consequently, a maximum grid size of 0.008 m was selected to maintain accuracy in the simulations. Furthermore, a consistent trend emerged, indicating that the calculation results obtained with the sliding mesh method were consistently lower than those obtained with the MRF model using the same mesh. A comparison of these two methods with experimental data is necessary to determine the most suitable approach. By evaluating their performance against experimental results, the method that aligns most closely with the observed data can be identified.

Accurately resolving the jet phenomenon in the external flow field of the extinguisher is crucial, necessitating a mesh independence check to determine the appropriate mesh size. The mesh data for various mesh sizes can be found in Table 2. For this study, a minimum inclination angle of 20° was selected, as it allows for the maximum jet distance and facilitates a comprehensive evaluation of the impact of grid size on jet continuity. Moreover, this angle serves to verify the mesh's ability to accurately capture the air speed near the ground. By carefully examining the mesh performance at this angle, the study ensures reliable and robust results regarding the jet behavior and the capability of the mesh to capture fine details near the ground.

**Table 2:** The external mesh data.

Mesh size (m)	Number of nodes	Number of elements
0.01	5,636,005	5,525,004
0.02	1,429,550	1,396,729
0.04	186,822	177,495
0.08	92,860	87,671

**Figure 7:**
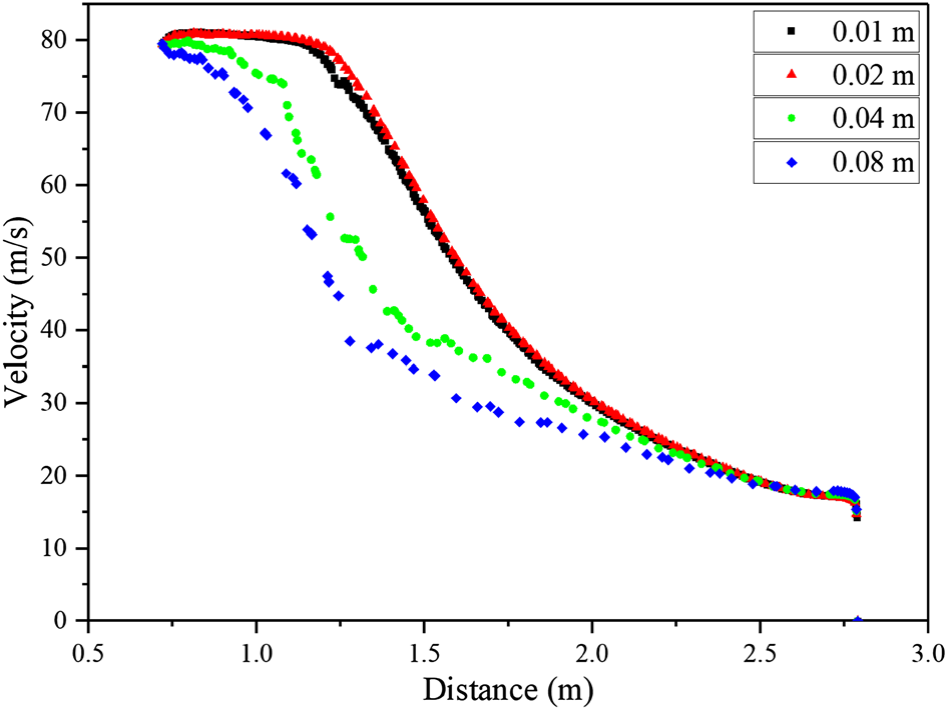
Comparison of the results calculated by different mesh sizes (External flow field mesh).

**Figure 8:**
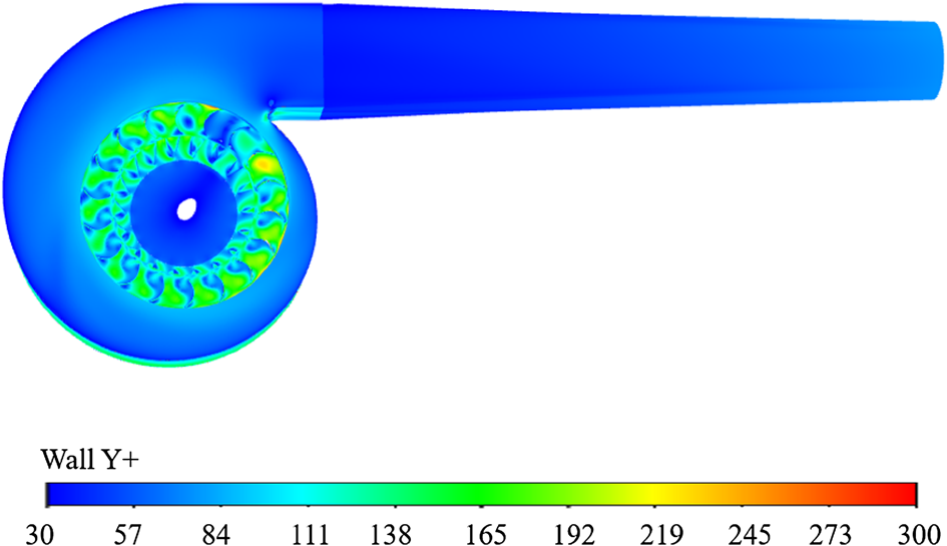
Distribution of Y+ values.

Once the calculation was completed, the velocity distribution along the central axis of the fan outlet was extracted and visualized in Fig. 7, with distances measured relative to the center of rotation of the fan. The figure revealed a significant deviation in the velocity distribution within the jet region when the mesh size was increased to 0.04 m. Consequently, a grid size of 0.02 m was selected to ensure more accurate representation of the velocity distribution. Additionally, Fig. 8 displayed the y+ values, demonstrating that the size of the first layer satisfied the calculation requirements of the turbulence model (30–300). This analysis confirms that the chosen grid size and first layer dimensions are suitable for capturing the turbulence characteristics in the computational model, ensuring reliable and precise results.

### Experimental validation

**Figure 9:**
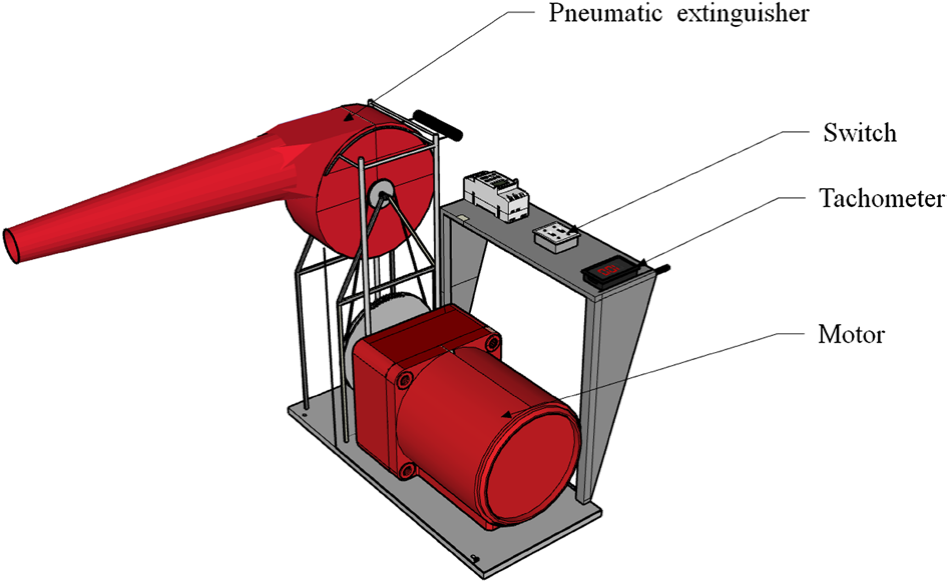
The experimental device.

In this paper, the air speed at the outlet of the extinguisher at the rated rotational speed and the velocity distribution of the jet field were measured separately to establish a reliable reference for the calculation results. The air speed was measured using a highly accurate Pitot tube. To ensure precise control of the impeller rotational speed, the gasoline engine inside the fan was removed and replaced with an electric drive. Furthermore, it was securely mounted on a stand to maintain a consistent height, as depicted in Fig. 9. In addition, rubber mats were installed at the bottom of the stand to minimize the noise generated by the experimental device. These measures ensured accurate and reliable measurements while reducing any potential sources of interference or disturbance.

**Figure 10:**
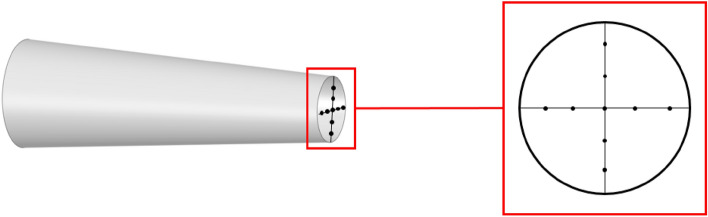
Measuring points.

**Figure 11:**
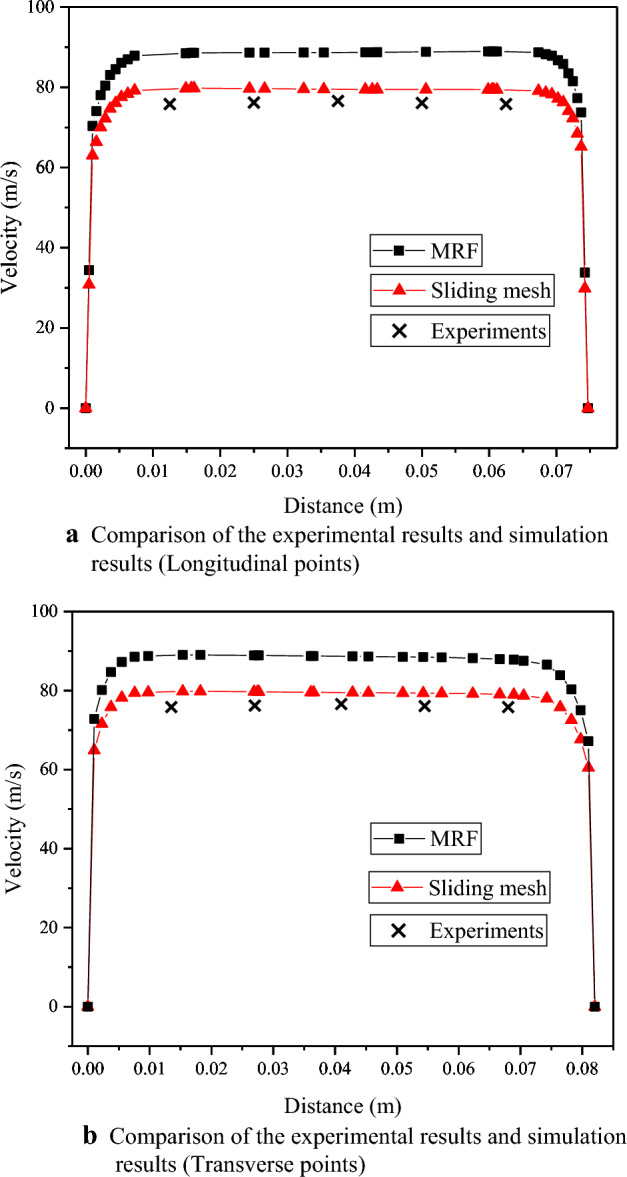
(**a**) Comparison of the experimental results and simulation results (Longitudinal points). (**b**) Comparison of the experimental results and simulation results (Transverse points).

Figure 10 illustrates the acquisition scheme for measuring the air speed at the outlet. Data is collected at five points horizontally and vertically, and a comparison is made between the measured data from these points and the calculated results. It is important to note that the values obtained from the Pitot tube measurements may exhibit oscillations within a certain range due to the inherent instability of the air speed. The average value within a 10-s interval is considered as the representative air speed at each point. The ambient temperature is maintained at 20 ℃ throughout the experiments. Figure 11a and b present the comparison graphs depicting the experimental and calculated results. The comparison reveals a significant discrepancy between the MRF calculation results and the experimental measurements, whereas the sliding mesh calculation results exhibit greater accuracy. The sliding mesh method allows for the creation of a grid that moves and rotates with the rotating domain, preserving the grid connectivity and maintaining a structured mesh. This feature enables the method to capture the rotational effects more accurately compared to MRF model. Consequently, the sliding mesh method was employed to conduct simulations of the internal flow field of the 6MF-30 pneumatic extinguisher.

**Figure 12:**
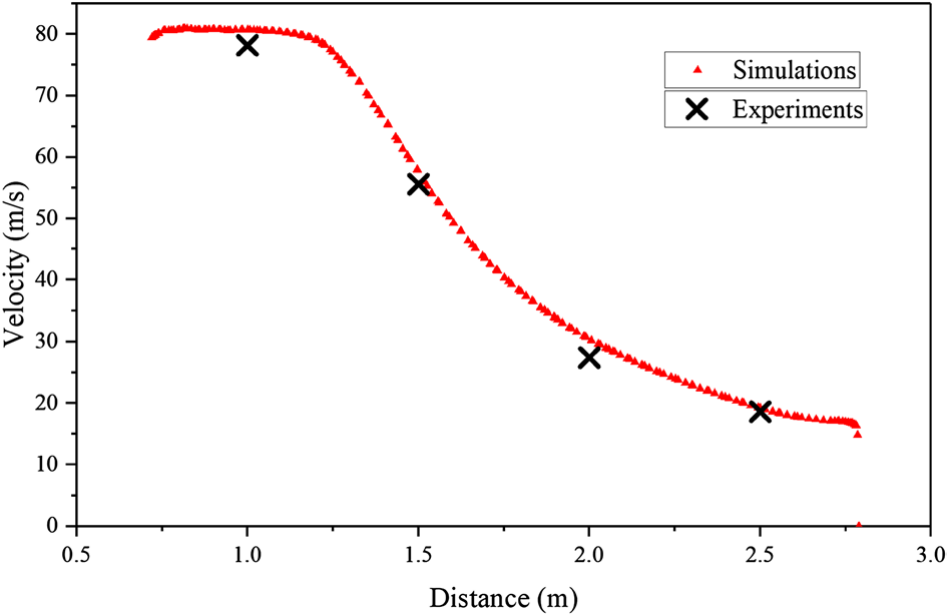
Comparison of the simulaiton results and experimental results (External flow field).

Four equidistant points were selected along the central axis of the outlet, located at distances of 1.0 m, 1.5 m, 2.0 m, and 2.5 m from the center of fan rotation. The air speed at each of these points was averaged over a 10-s interval to obtain the average speed value. Figure 12 displays the comparison graph illustrating the experimental and simulation results. The graph indicates a close correspondence between the simulation results and the measured values, indicating the reliability of the calculated results as reference data.

## Results and analysis

The previous analysis has determined an appropriate mesh size and demonstrated the reliability of the calculation results as reference data. To ascertain the fire-extinguishing range and determine the optimal inclination angle of the portable pneumatic extinguisher at its rated rotational speed, this study considered the critical air speed for fire extinguishing specified in *the National Standard GBT 10280-2008 for Forestry Machinery Portable Pneumatic Extinguisher*, which is set at 22 m/s. Consequently, the area close to the ground, where the air velocity exceeds 22 m/s, was chosen for calculating the projected area, conducting comparisons, and analyzing the fire-extinguishing range at various inclination angles to ascertain the optimal inclination angle. Furthermore, this paper conducts a comparative analysis of the velocity distribution of the jet under different inclination angles. The detailed calculation results and analysis process are presented below.

### Optimal fire extinguishing angle determination

It is important to clarify that the findings regarding the extinguishing range and optimal inclination angle discussed in this paper specifically apply to grassland fires occurring in flat terrains. These grassland fires exhibit characteristics such as low vegetation height, uniform distribution of combustible materials, absence of humus accumulation on the surface, and a reduced likelihood of overcast combustion. These traits align with the typical scenarios in which the 6MF-30 portable pneumatic extinguisher is employed.

#### The best inclination angle on the lossless ground

**Figure 13:**
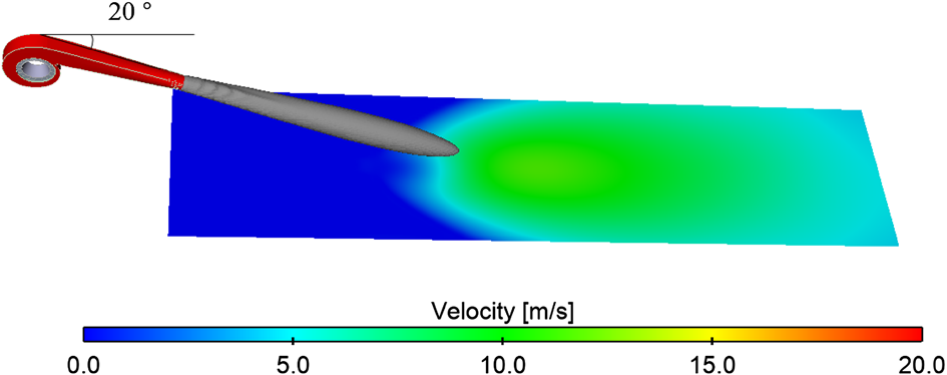
The 22 m/s equivalent surface of the calculated results for the inclination angle of 20°.

**Figure 14:**
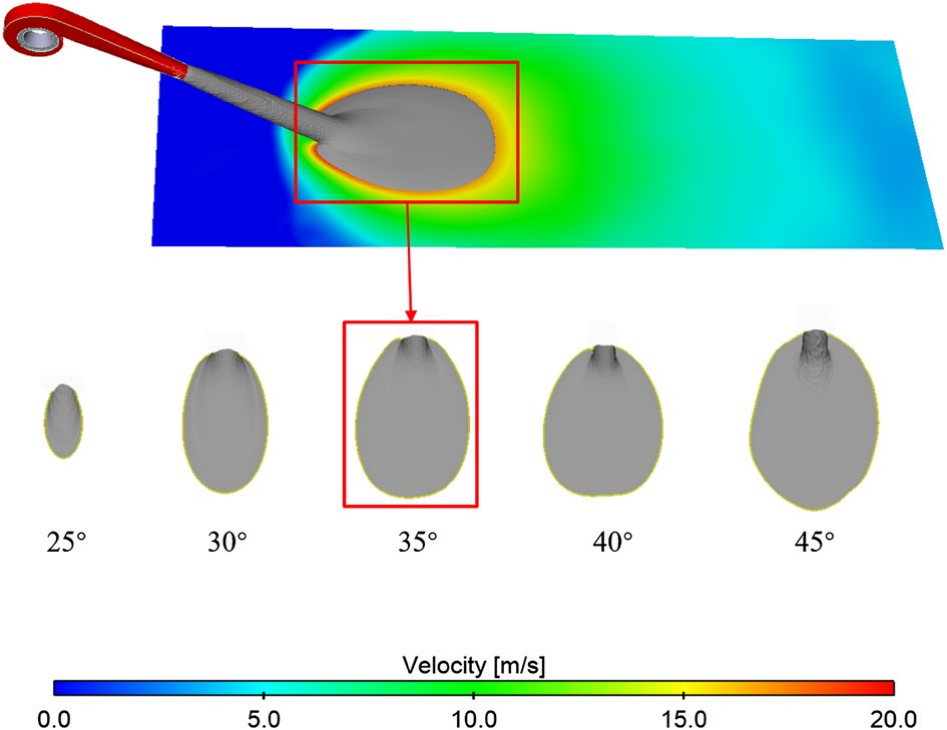
The scheme of protective area comparison.

**Figure 15:**
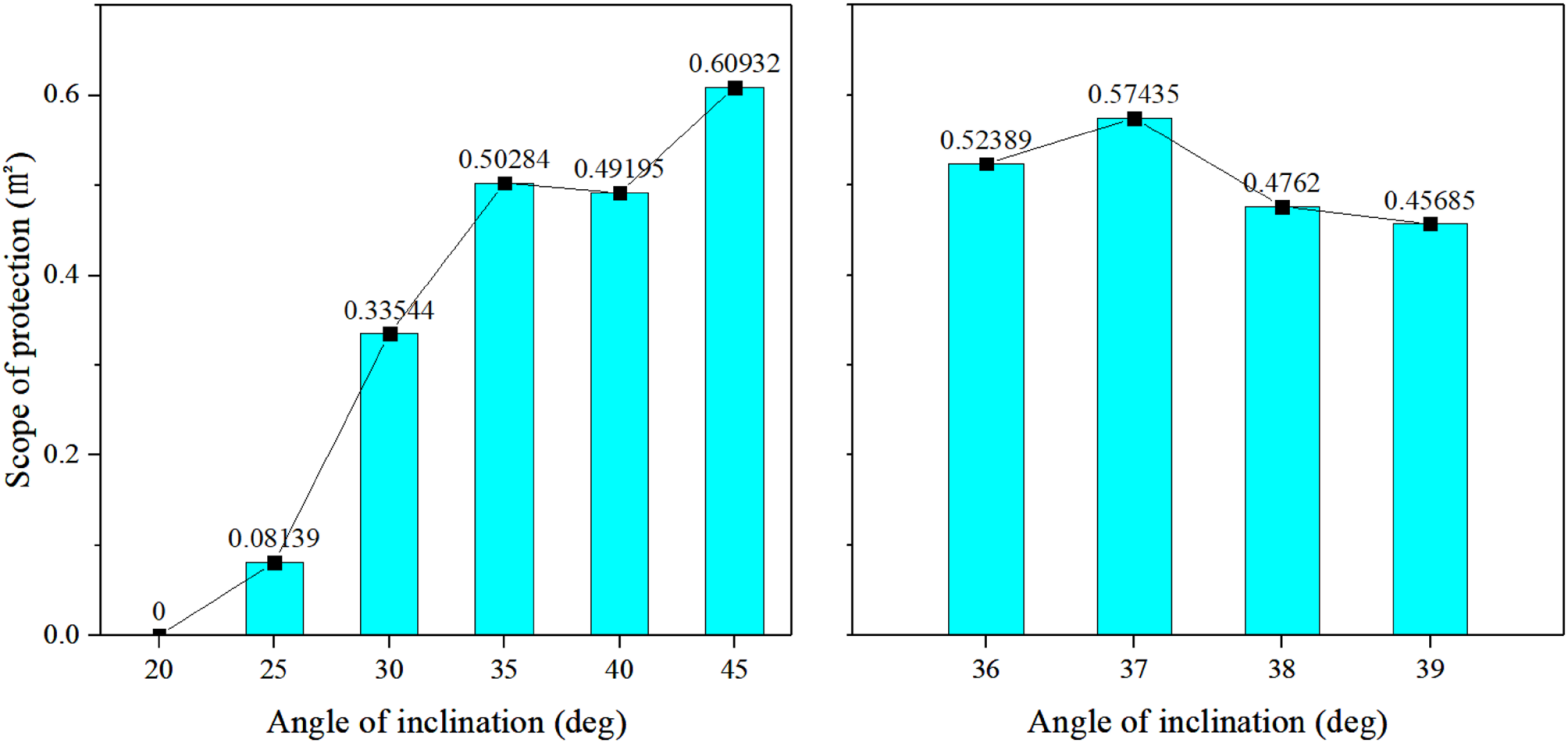
Comparison of the scope of protection from different angles of inclination.

Initially, the simulation assumes an ideal scenario with a smooth ground surface, disregarding any roughness effects. Figure 13 illustrates the equivalent surface at the inclination angle of 20° for the calculated results with a velocity of 22 m/s. Observing the results, it is evident that the jet remains above the ground at the inclination angle of 20°, with a near-ground airflow velocity below 20 m/s, resulting in a near-ground protection area of 0. Figure 14 presents a top view of the near-ground equivalent surface for the remaining working conditions. Figure 15 provides a comparison of the protection areas, clearly illustrating that the largest protected area corresponds to the 45° working condition, while the smallest is observed under the 25° working condition.

However, as the inclination angle increases, the extinguisher gets closer to the fire source, necessitating a careful consideration of both the extinguishing range and the proximity to the fire source when selecting the optimal inclination angle. The 45° inclination angle brings the extinguisher closest to the fire source, but the protection range under 40° is smaller than that under 35°. Hence, this paper explores the 30° to 40° range to determine the optimal inclination angle. Initially, the study calculates and analyzes the protection range for the middle values within the intervals of 30° to 35° and 35° to 40°, specifically 32.5° and 37.5°. The resulting protection ranges for these two working conditions were 0.34 m^2^ and 0.56 m^2^, respectively, leading to a narrower interval of 35°–40°. The study further explores the working conditions at 36°, 37°, 38°, and 39°, presenting the calculation results in Fig. 15. Observing Fig. 15, it is evident that the largest protection area, measuring 0.57 m^2^, is achieved at an inclination angle of 37°, making it the optimal fire extinguishing angle.

#### Determination of the best inclination angle in a typical grassland scenario in the Xilin River basin

**Figure 16:**
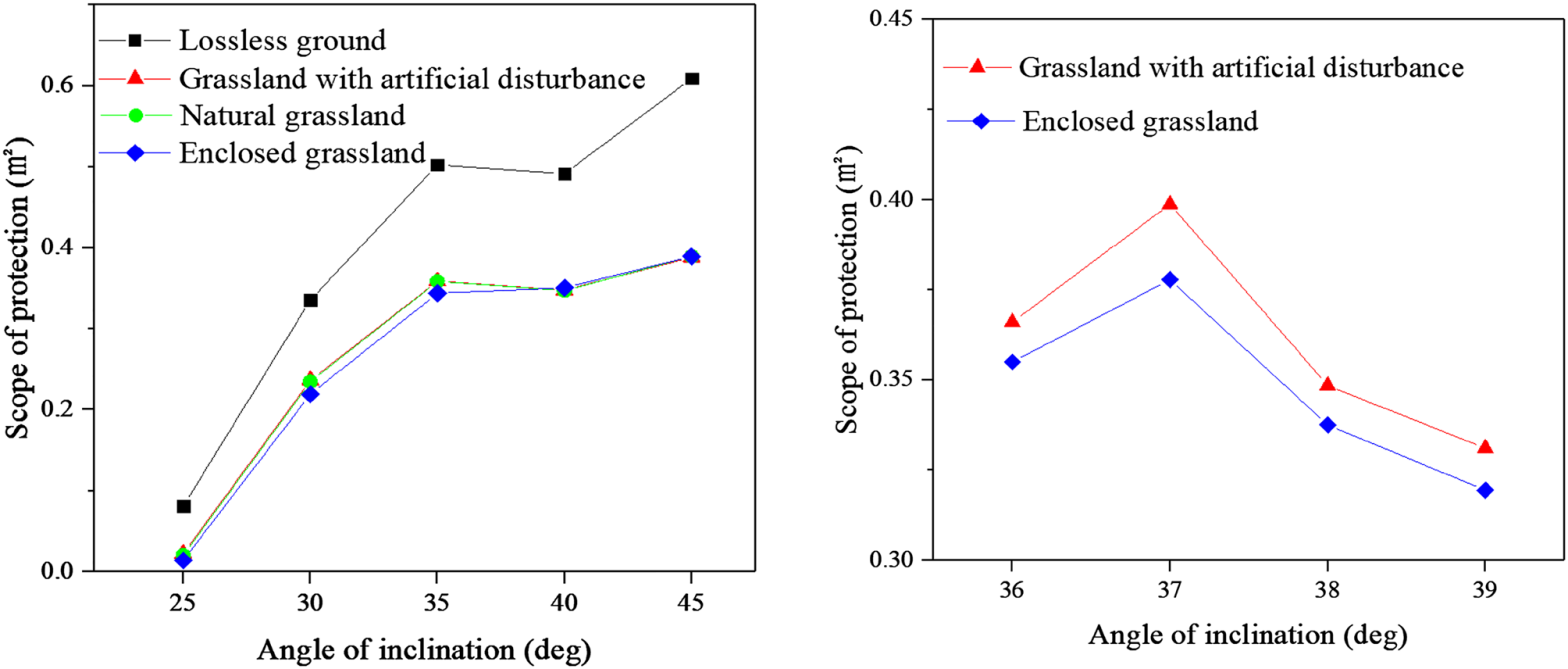
Comparison of the scope of protection from different types of ground.

However, real grassland scenes have uneven ground surfaces with varying degrees of roughness. Zhang Qi et al., classified the typical grassland types in the Xilin River basin into three categories: natural grassland, enclosed grassland, and grassland with artificial disturbance. Their experimental findings reveal variations in roughness among these grassland types, with enclosed grassland exhibiting the largest surface roughness of 0.0268 m, followed by natural grassland at 0.0193 m, and grassland with artificial disturbance at the smallest surface roughness of 0.0184 m^30^. The ground boundary wall roughness was defined in ANSYS Fluent based on the aforementioned analysis. The fire extinguishing ranges for each of these three grassland types were investigated individually, and the specific results are presented in Fig. 16. The calculated results for the inclined 20° working condition are not compared in the figure, because in the ideal case, the near-ground protection area for this condition is 0. Even after considering ground roughness, the portion of its jet above 22 m/s still does not reach the near ground.

Figure 16 illustrates that the protected areas of the three typical grasslands exhibit minimal differences, with only the calculated results for the enclosed grassland at inclination angles below 35° slightly lower than the other two grasslands. At identical inclination angle, the protection area of the three typical grasslands exhibits a considerable decrease compared to the lossless ground, with the difference increasing as the protection area expands. However, the trend remains consistent, except for the calculated results at 35° for the enclosed grassland, which are slightly smaller than those at 40°. Based on the figure, it can be inferred that the optimal inclination angles for the three typical grasslands also fall within the range of 35°–40°. Within this range, the curves for grassland with artificial disturbance and natural grassland exhibit similar patterns, allowing them to be grouped together. Therefore, inclined conditions of 36°, 37°, 38°, and 39° can be selected in combination with the enclosed grassland. The corresponding calculation results are presented in Fig. 16, indicating that the largest protection range is achieved at an inclination angle of 37°.

In conclusion, the optimal inclination angle of 37° applies to both lossless ground and all three typical grassland scenarios. The analysis process reveals that there is no substantial correlation between the optimal inclination angle and the ground's roughness. Thus, the optimal fire extinguishing angle of 37° is equally effective in scenarios with varying levels of ground roughness.

### Effect of inclination angle on the jet velocity distribution

**Figure 17:**
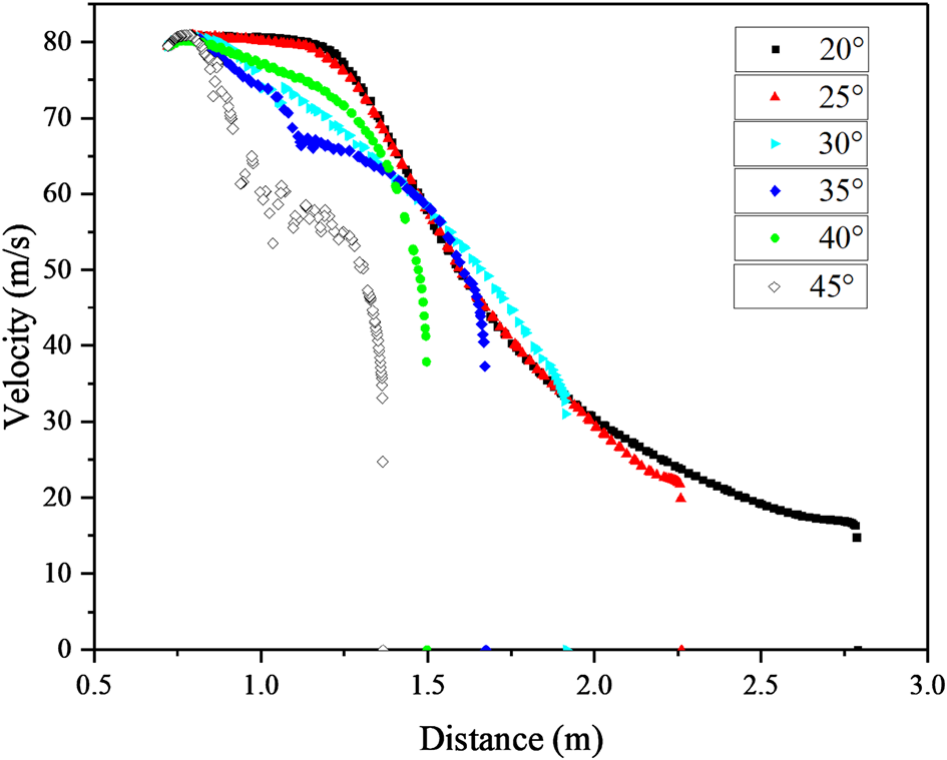
Comparison of the jet velocity distribution from different angles of inclination.

Figure 17 illustrates the velocity distribution along the mid-axis of the outlet for all operating conditions above the lossless ground. The figure demonstrates that the jet velocity near the extinguisher outlet decreases most rapidly at a 45° inclination angle, followed by 35°, 30°, and 40°. In comparison, 20° and 25° exhibit similar rates of decrease and the slowest reduction among the six operating conditions. Consequently, it can be inferred that when tackling wildfires involving combustible materials like brush with a specific height, a larger inclination angle does not necessarily result in improved fire suppression. Instead, a smaller inclination angle can generate a higher air jet speed at the same location.

**Figure 18:**
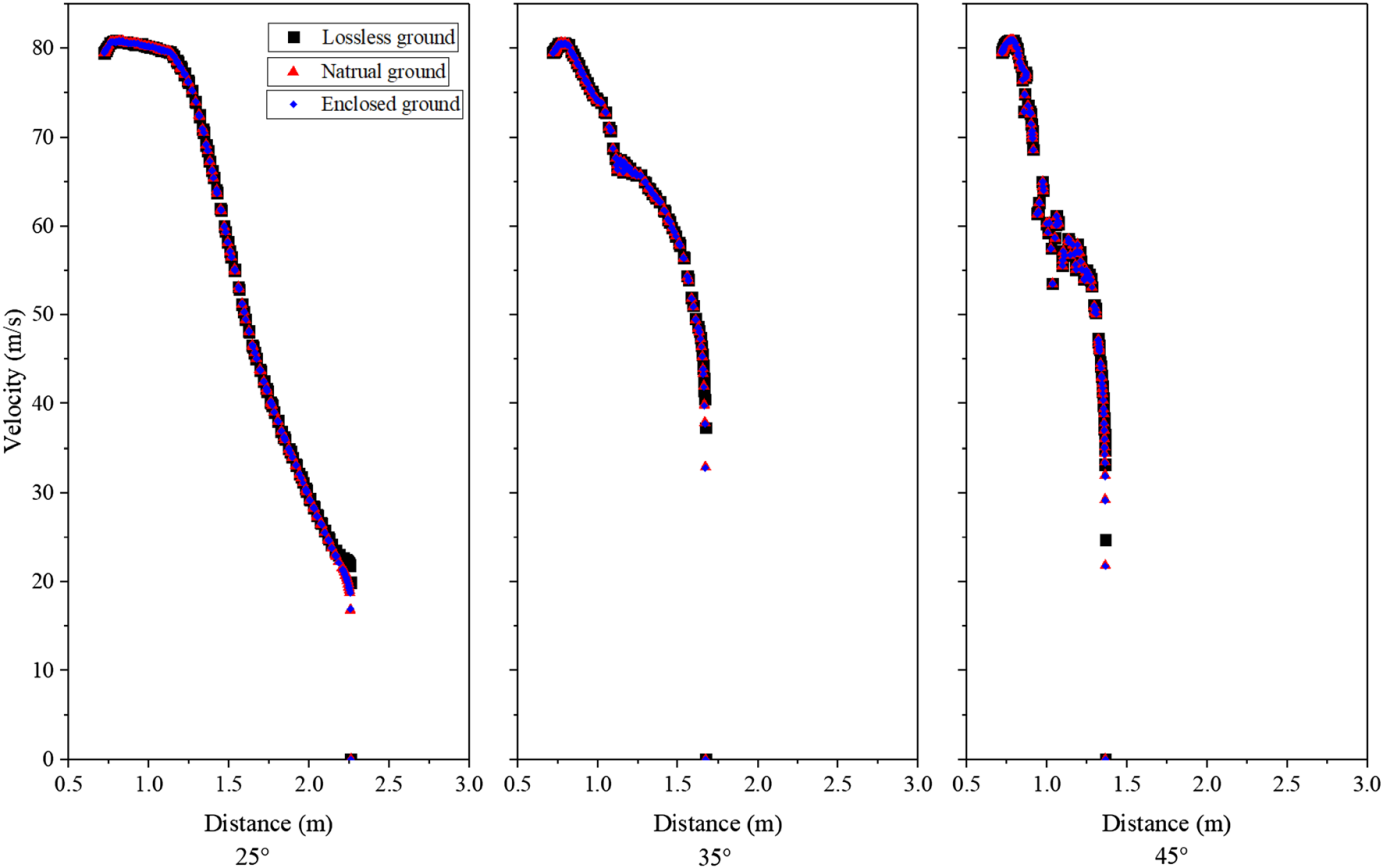
Comparison of the jet velocity distribution from different roughness.

Based on the distribution of jet velocities, the six conditions depicted in the figure can be classified into three categories: the 20° and 25° conditions exhibit similar curve shapes and experience the slowest rate of decay near the fan exit area, the 45° condition demonstrates the fastest decline near the outlet region, while the remaining conditions belong to a separate category. To compare the decay of jet velocities in different grassland types, these three specific groups of conditions were chosen as representatives of 25°, 45°, and 35°, respectively. The comparative results are presented in Fig. 18. The figure indicates that the three sets of curves closely overlap at the same inclination angle, with only variations observed in the near-ground area. This observation suggests that the impact of ground roughness on the decay of jet velocity can be disregarded, and that the inclination angle alone has a significant effect on the distribution of jet velocity.

## Conclusions

This study employs computational fluid dynamics simulation and experimental verification to analyze the protection range of the 6MF-30 pneumatic extinguisher at its rated rotational speed by considering multiple groups of inclination conditions. The key findings of this paper are summarized as follows:

1. The MRF method yields higher air speeds at the fan outlet compared to the sliding mesh method, while the calculation results of the sliding mesh method exhibit closer agreement with the experimental data.
2. The optimal extinguishing angle for both the lossless ground and the three typical grassland grounds is determined to be 37°. Interestingly, the ground roughness does not significantly impact the optimal inclination angle of the extinguisher. The largest protective area is observed under a 45° angle condition, which also corresponds to the closest proximity to the fire source. Notably, the protective area under a 37° condition is comparable to that under the 45° condition, thus leading to the selection of 37° as the optimal extinguishing angle.
3. The inclination angle significantly influences the velocity attenuation of the jet along the central axis of the outlet in the vicinity of the fan outlet. Specifically, the velocity attenuation is most rapid at 45°, while it is slowest at 20° and 25°. Notably, the velocity attenuation under 40° is slower than that under the 30° and 35° conditions. Furthermore, the effect of ground roughness on this phenomenon is not evident.

## Data Availability

The datasets generated and/or analysed during the current study are available from the corresponding author on reasonable request.

## List of symbols

$k$: Turbulence kinetic energy
*Z*_0_: Ground roughness
*Z*_1_, *Z*_2_: Altitude
*U*_1_, *U*_2_: Airflow speed at *Z*_1_ and *Z*_2_

## Greek

$\varepsilon$: The turbulence dissipation rate
$\omega$: The specific dissipation rate

## Subscripts

CFD: Computational fluid dynamics
MRF: Multiple reference frame
